# Simulation of Glacial Avalanche Hazards in Shyok Basin of Upper Indus

**DOI:** 10.1038/s41598-019-56523-7

**Published:** 2019-12-27

**Authors:** Naseem Gilany, Javed Iqbal

**Affiliations:** Institute of Geographical Information System, School of Civil and Environment Engineering, National University of Science and Technology, Islamabad, Pakistan

**Keywords:** Climate sciences, Natural hazards

## Abstract

Glacial avalanche hazards can threaten lives and damage infrastructures in high altitude mountainous regions. In April 2012 a gigantic ice plus rock avalanche destroyed military camp at Gyari and killed 139 persons. Antecedent, the objectives of this study are to simulate and model Gyari camp glacial avalanche with reference to its extent, height, momentum, velocity and pressure and, to simulate and model the other potential glacial avalanche prone areas in Shyok basin. To simulate the Gyari camp glacial hazard and other potential glacial avalanche hazards, an empirical process based Glacier Avalanche Model; Rapid Access Mass Movement Simulation (RAMMS) is used. The RAMMS model encompasses the variables like avalanche release height and release area for the conduct of simulation. The model output of Gyari glacial avalanche hazard resulted from a max pressure of 2500 KPa, max velocity of 90 m/s, and the max flow height of 40 m, while the resulted output debris volume calculated was 4.3145 million m^3^. The calibrated agreement was found in extent and height of actual debris in comparison with RAMMS simulated output. The potential hazardous glacial avalanche prone areas of Shyok basin were simulated by RAMMS model after the model being calibrated to the actual incident of Gyari. The study has resulted in identifying the Siachen glacier conflict zone being more prone to avalanche hazards because of host factors in general and the anthropogenic factor in particular.

## Introduction

Glacial avalanche is the critical hazard which effects buildings, roads and poses threat to humanity in mountainous regions. Ice avalanche usually occurs, once a huge mass of ice detaches from a limp glacier. Human lives and structures have been affected by avalanches in Alps, Kashmir, Iceland, Afghanistan, Caucasus and Canada^1^. The ice avalanches are the indicators of the regular depletion phenomenon of the steep glaciers in higher elevation ranges having high relief energy. When the glacial avalanches are coupled with debris, then extreme disasters are caused by these avalanches^2^. On 07 Apr 2012 in Siachen sector of Pakistan, a military camp situated in Gyari suffered from a massive glacial avalanche resulting burial of 139 soldiers and civilians under a huge mass of debris/snow.

### Avalanche types

Avalanches have two broad categories; loose snow avalanche and slab avalanche.

#### Loose snow avalanches

Loose snow avalanches are common on sheer slopes and comparatively less damaging. They constitute less apparent layers and commonly shallow snow. Loose snowfalls can be both dry and wet. These are able to conceal an individual.

#### Slab avalanches

Slab avalanches happen on sheer slopes; these are cavernous, inflexible and able to spread over long distances. These are the most destructive type. Their utmost unique feature is a spotless stretchy fracture at the top (at the crown). The release of a slab avalanche needs the failure of a weak layer or weak boundary beneath snow lump. The delicate nature, insistent weak layers and the boundary overhead are often problematic to distinguish and are the reasons of most avalanche mortalities.

Classification of weak layers depends upon the processes which cause the formation of the grain type. These are named as depth hoar, surface hoar and facets types.

Slab avalanches have several parts, the “crown”, usually it comprises of a break mark at the higher limit on the slope, the “flanks”, which are continuation of the crack lines down together on each side of the slab, the “staunch wall”, the lowest limit of the slab; which is frequently demolished as the avalanche advances down the slope and the “bed surface”, at which the avalanche slips; which is generally horizontal and planar, though it can be the ground itself.

### Avalanche formation and release

Snowpack features, meteorological elements, and terrain structures are important in prompting snow strength, avalanche relief, and avalanche indication along with the run-out. The complex dealings of these dynamics that initiate avalanche release are mentioned as avalanche formation. To measure snow strength, avalanche experts look for the existence of weak strata surrounded by the snowpack.

Meteorological elements influencing strength comprises of air temperature, wind, relative humidity, solar radiation, long wave radiation, snow accumulation rates, snow depth, and rainfall. These elements subsidize the development of weak strata inside the snowpack. Moreover, fresh precipitation ahead of a snowpack might activate a weak stratum to apart an avalanche.

### Statistical parameters of glacier avalanches

In general, the glacier associated hazards assessment process is often based on understanding of simple basic knowledge and capabilities extended for past events. Instead of separate processes, an integrated overview of the detailed investigations of the chain reactions and glacier avalanche disasters are required. The numerical parameters concerning initial conditions of starting and accumulative deposition of the ice avalanches are mainly the resultant of case studies by Alean in the Swiss Alps which are discussed as under.

#### Avalanche initial environment

The preliminary situations according to Alean rest on the type of failure. Ice discontinuation might be the consequence of morphological categories like ramp type and cliff type glaciers regions of possible ice avalanche. Ramp type glaciers are located on relatively unvarying slope regions in comparison with cliff type glaciers. Perhaps as a consequence of the surroundings temperature rise, the dangers of ice avalanche detach rises with the increase in temperature of inside and at the bed of the glaciers.

#### Slope and ice temperature

Slope coupled with ice temperature is mainly accountable for nature of the avalanches. Glaciers having bed slope within range of 25° to 45° create avalanches as mentioned in Table 1. Even though ice glaciers lean towards to be additional steady, it is sensible to think through glaciers being steeper than 25° as possibly hazardous. According to avalanche specialists all over the world after suitable meteorological conditions, the topographical slope in an essential aspect in understanding and forecasting possible avalanches.

**Table 1:** Slope and avalanche probability.

Slope	Avalanche Probability
10°	Practically no avalanches are triggered
10°–28°	Avalanches are scarce
28°–45°	The major danger zone for avalanche triggering
45°	High avalanche frequency, however low snow accumulation due to the steepness

#### Cliff type preliminary zones

Cliff type initial regions are situated at a point wherever a noticeable rise happens on the slope of the topography. As the glacier grows an approximately vertical façade, the detachment process for this kind is very likely. The glacier bed inclination has a certain association with the occurrence of avalanches. Because of the great stretch and stress near the vertical facade, ice breaks into small pieces.

#### The run out distances

The track related to the detachments of ice-rocks falls have extended up to 6000 m in the European Alps, with even complex values identified by additional mountain ranges. On behalf of a major valuation founded on these factually recognized extreme run out detachments, the zone possibly confronted by the main avalanche be approximately defined like zone surrounded by the extreme spread.

### Remote sensing application in avalanche studies

At present, the identification and representation of avalanches depend mainly on the way observations attained by professionals on ground situations. Therefore, the analyzed region is relatively poor as individual measures inside a limited area. To a certain extent, the avalanches consequential in heavy reimbursements are recorded a little. Most of the times there are huge areas which are unreachable to viewers, particularly if the avalanche hazard category is high. Efficient identification and recording of avalanches over huge areas are very vital; the attainment of such statistics would be significant for sovereign assessment of the accurateness of avalanche reports.

Remote sensing mechanisms are capable of getting information in excess of large regions deprived of limitations produced by poor ground convenience^3^. The remote sensing is used to get the information of snow cover attributes like snow depth, water content, and diameter of snow particles^4^. By utilizing Digital Terrain Model (DTM) of the glaciated areas the steepness is observed and, the identification of glaciers can be done by band combination of spectral data^5^. The DTM has been utilized for the modeling of glacier avalanche hazard assessment. Temporal, high resolution and accurate remote sensing data allow observation of mass variations involving kinematics^6^.

### GIS application in avalanche studies

The spatial analysis accompanied by modeling abilities of Geographic Information Systems (GIS) has the ability to estimate parameters of glacier avalanche-like debris extent, flow height, velocity, momentum, and pressure. GIS has the capability to develop avalanche Spatio-temporal variations by examination and visualization of avalanche topography. Advancements of web-based software compatible with GIS has made a step ahead in order to make awareness to the communities on possible avalanche threats. A common practice of using Geographic Information Systems (GIS) in avalanche research studies is followed in order to extract terrain attributes for analysis of the formation and triggering phenomenon of avalanches^7^. By using GIS the avalanche release zones can be defined by analyzing aspect, slope, and proximity and elevation curves from Digital Elevation Model (DEM)^8^.

### Rapid access mass movement simulation (ramms) glacier avalanche model

A precise estimation of glacial avalanche flow velocity, flow height, influenced pressure and run out distances in the normal 3D environment; is carried out with the advance glacier avalanche model; RAMMS. The model is set up by Swiss Federal Institute for Snow and Avalanche Research^9^. In avalanche replications, the method of hyperbolic rules is the base of mean depth avalanche models. In order to make an assessment of the avalanche hazard extent, 1D mathematical model, such as AVAL-1D is used in Switzerland, America, Europe and Asia^10^. The avalanche flow size and flow course are to be marked by the user in 1D model. 2D and 3D models require a complete procedure to fulfill the topography requirements and release parameters. However, the handling of flow rheology is the major problem in all these 1, 2 and 3D models.

In RAMMS two different models like Voellmy Salm (VS) and Random Kinetic Energy (RKE) are utilized to deal with the flow rheology. The statistical model is nearly realistic in order to estimate the extents of potential hazard-prone areas in future and for the determination of past known avalanche happenings at specific locations.

### Background

Gyari, a vital military base of Pakistan armed forces was located in a deep valley of the Siachen glacier warzone, at an elevation of 3775 m. On 07 Apr 2012, a huge ice plus rock avalanche hit the Gyari base camp. It was an important supply center of troops and material to be passed to further remote sites. The glaciated avalanche struck one of the trained battalion in mountainous terrain which was operating in world’s highest battle zone^11^. Avalanches were rare in Gyari sector which was a larger compound and also contained a huge contingent of the army to safeguard the other camp sites in the Siachen zone. At an elevation of 4560 m from mean sea level, the avalanche arose from glacier at around 1.3 kilometers away from camp. The Pakistan Army had stated all the fatalities of Gyari sector as ‘Shuhada’ (martyrs), as nature and scale of the disaster was evocative so the probability of recovering anyone alive was almost impossible.

The current study explored the capabilities of remote sensing (RS), geographic information system (GIS) and physically based glacier avalanche model; RAMMS to identify potential initiation zones and discover the spatial distribution of possible ice avalanches, debris flows, avalanches velocity, pressure and momentum for hazard assessment in Shyok basin of Pakistan.

### Objectives

The objectives of the study are as following:-

1. To simulate and model Gyari glaciated avalanche by utilizing RAMMS model in order to assess flow height, velocity, extent, momentum, and pressure generated.
2. To make an assessment of potential hazard risk of other glacial avalanche prone areas in Shyok basin through modeling & simulation.

## Materials and Methods

### Study area

**Figure 1:**
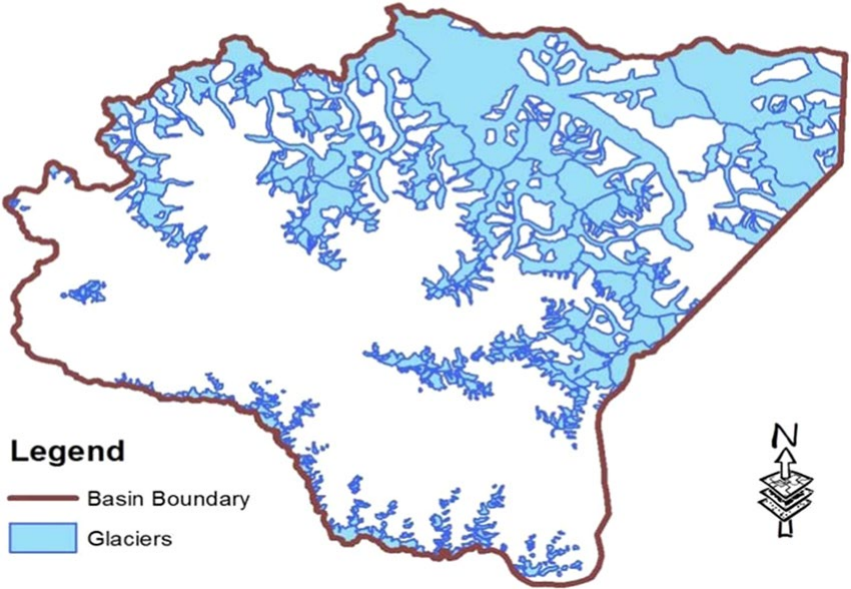
Glaciers of Shyok basin.

The study area comprises of the Shyok basin (Fig. 1). The basin contains Siachen glacier, one of the largest glacier outside the Polar Regions. The study area has also an importance from the military point of view as two main military camps are situated here. The Gyari sector is situated in the Shyok basin which contains many of the hazardous glaciers.

The living environments at Siachen are so harsh that if naked skin exposes to ice, it gets frostbite.

### Data and materials

Following datasets were utilized for the study:-

#### Remotely sensed satellite data

In present study, the pre-disaster and post-disaster GeoEye high-resolution satellite imagery of the Shyok basin is utilized. Landsat 30 m resolution satellite images were used for detailed point snow cover.

#### Digital elevation model

The basic input for modeling glacier avalanche studies is considered as Digital Elevation Model (DEM). In this study, ASTER GDEM of 28 m resolution covering the whole region is incorporated.

### Analytical framework

**Figure 2:**
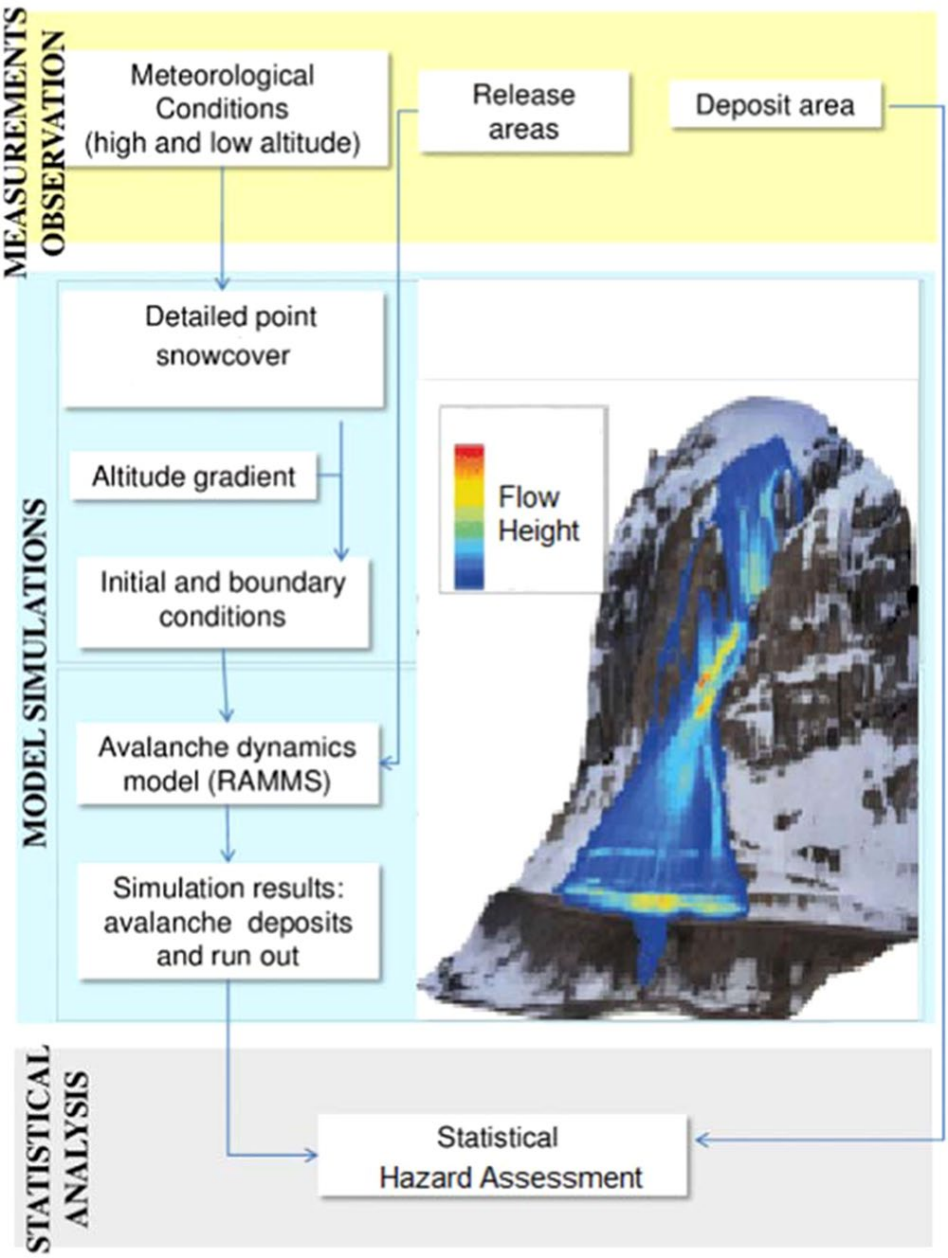
RAMMS model workflow (http://ramms.slf.ch).

The methodological flowchart for the RAMMS model is as shown in Fig. 2.

The methodology adopted for the study is as following:-

1. RS and GIS data preprocessing.
2. Calibration for validation of RAMMS; glacier avalanche model for simulation of Gyari glacier and other potentially dangerous glaciers in the study area.

### Data preparation

#### Pre and post disaster satellite image

For effective use of remotely sensed satellite data, a number of pre-processing techniques were applied on it prior to analysis. For pre and post disaster imagery, GeoEye Google Earth image was acquired using Google Earth Pro and the images were downloaded at high resolution. All the high-resolution scenes were rectified and projected individually in Universal Transverse Mercator (UTM) projection system and reference datum WGS-84. After rectification, all high-resolution tiles of GeoEye satellite were mosaicked and the resultant was a high-resolution GeoEye imagery of study area. Following pre-processing steps were performed on pre-disaster imagery before using the image for GIS analysis.

- GeoEye Google Earth data is a high-resolution image with 60 cm spatial resolution; each high-resolution image tile was mosaicked to form a single high-resolution image. After mosaicking, a full high-resolution image of the study area was obtained.
- Image sub-setting was performed to extract the area of interest from the mosaicked image using the boundary of the study area.
- There was no need to apply atmospheric correction on the Google Earth imagery because it was already preprocessed. However, after sub setting, an image stretch was automatically performed on it so that the image could be de-stretched.

All these pre-processing tasks were performed using utilities in ERDAS IMAGINE 2011 software.

#### Digital elevation model utilization

Digital Elevation Model (DEM) is used to characterize the several high mountain geomorphologic processes which are obsessed by the relief dynamism. In addition, DEM is required for ortho-rectification of image files, for precise geo-referencing and for co-registration.

ASTER 28 m DEM was incorporated for the analysis. In the preprocessing of DEM, the sinks were identified and then those sinks were filled using ArcGIS software. After sink/fill operation the slope, aspect, flow direction, and flow accumulation was calculated for the Shyok basin.

#### DEM verification

There are numerous techniques to examine the exactness of Digital Elevation Models (DEMs) produced by taking measurements from the satellite images. These are comprised of:-

- Creation of contours or shaded relief to permit for a pictorial review of irregular topography.
- Examination of frequency histograms of main topographical features.
- Measures of residuals during processing.
- Evaluation of height values from the DEM to measured height points on the ground.

The ASTER GDEM was compared with the surveyed data and it was found that it can be used significantly. The accuracy of ASTER 28 m GDEM at Gyari avalanche debris site was obvious as ASTER data points were in a systematic manner while surveyed points were random. There was a difference between ASTER data points and the surveyed points on the far side of the camp, which was because of the fact that there was almost a vertical cliff present at that side.

### RAMMS avalanche model inputs

RAMMS; a computer-based model for simulation of glacier avalanche is highly reliable. The model has been calibrated for verification with a number of happenings of avalanches. The model outputs are used for estimation of flow velocities, flow heights, run out distances and influence forces. Geo-referenced maps or airborne images can also be incorporated into RAMMS. The input parameters required to accomplish the mathematical scheming of RAMMS are as shown in Table 2.

**Table 2:** General inputs required for RAMMS.

Ser.	Inputs
1.	Digital Elevation Model
2.	Fracture Height and Release Zone
3.	Friction Parameters
4.	Avalanche Return Period
5.	Avalanche Volume Category
6.	Snow/Glacier Density
7.	Forest Parameters
8.	Aerial/Satellite Imagery
9.	Domain Area

#### Digital elevation model

All statistics related to the complications and geometry of the regular topography is calculated from DEM. The DEM spatial resolution has a main influence on the avalanche trail and course dynamics.

The DEM resolution which describes the normal topography is the utmost significant contributing factor. The glaciers melting behavior is associated with altitude gradient. At an altitude of >14000 ft, glaciers are stable and at an altitude of <14000 ft, glaciers are more prone to glacial hazards. An accurate simulation includes a high-resolution input DEM, e.g. a 5 m DEM resampled from 10 m or maybe 10 m from 20 m^12^. For the current study, ASTER 28 m corrected GDEM was incorporated into RAMMS.

#### Fracture height and release zone

Release zones were identified by means of polygon shape files created in RAMMS model. The outcomes of GIS designed topographical examination can be incorporated in RAMMS. The fracture height and release zone areas are necessary for huge scale hazard representing applications with RAMMS.

#### Friction parameters

By conducting an extensive terrain analysis, friction parameters can be prescribed, based on the procedural classification of terrain features into categories as open shape or flat terrain, channeled or gully and forested or non-forested. For the present study analysis, the friction parameters are kept constant for the domain area input to RAMMS.

#### Avalanche return period

The avalanche return period was pre-defined as 10 years, 30 years, 100 years and 300 years. For this research study, it was adjusted to 30 years after a long time of model results.

#### Avalanche volume category

The model did not take into account the volume category in terms of quantity; rather it was qualitative as tiny, small and large. The avalanche volume category was set to large for the present study.

#### Snow/glacier density

One of the inputs to the model was the density of the material present at the settlement sites. Currently, the density of 917 kgm^−3^ was set being the density of the glacial mass. As the avalanches under study start in cold conditions (snow-cover initial release temperature and entrainment cold snow temperature). Therefore may not change phase under most topographic condition. Avalanche temperature, however, not only depends on the initial and boundary conditions, but also on the path-dependent frictional processes that increase internal heat energy. RAMMS account for the processes: Frictional shearing in the slope-parallel flow direction and Dissipation of random fluctuation energy by inelastic granular interactions. In avalanche flow, nonlinear irreversible processes are coupled with RAMMS input variables: initial and boundary conditions that lead to transitions in flow regime. Redistribution of snow by wind is a major feature of mountain snowpack and it is essential for avalanche formation and density. Snow avalanche happens in extreme weather because of redistribution on blowing and drifting snow^13^. Various natural geologic hazards can be induced by non-uniform distribution snow layer caused by drifting snow, and it can contribute greatly to the mass balance of the ice sheets. The coupling effect of turbulent wind on saltation snow particles using a Lagrangian dynamic subgrid-scale model has been observed as one of the cause of snow avalanche^14^.

#### Forest parameters

The model takes the forest cover present at the site of the avalanche into account. Forest cover is also a significant parameter as it provides resistant to the avalanche trail and slowdowns its impact. But in this research study, there was no forest cover present at the study site so no forest input was incorporated into the model.

#### Aerial/satellite imagery

The Landsat 30 m resolution satellite images were used for observations to calculate the snow-cover property. The Geo Eye satellite images were used in the model to visualize the simulation results and to identify the important inputs like release area, forest cover, and the impact of the avalanche hazard in the Shyok basin.

#### Slope calculation

The slope was derived from the digital elevation model. The slope was used to assess the layout of the topography in Shyok basin. The slope values in the basins vary in range from 0° to 72°. The slope of 0° was found almost parallel to the river beds whereas the slope of 72° was observed near the mountains and cliffs.

### Glacier avalanche simulation

The avalanche risk modeling of all the glaciers in Shyok basin were done using RAMMS model. To achieve this purpose all the inputs, mentioned in Table 2 were incorporated in the RAMMS model. The model was run for every single glacier at a time with the preprocessed inputs.

The output of modeled glacier avalanches was coupled in the ArcGIS environment. The trails of avalanches in Shyok basin in terms of debris extent were prepared. This was done to eliminate the area which had direct exposure to the avalanche. The glacier avalanche was modeled for their worst conditions to cater for the max area under risk.

## Results and Discussions

In most of the regions of the world, especially where there is poor documentation of natural hazards, the destruction caused to human lives and properties by glacial avalanches remain underestimated. Siachen conflict zone is one of the regions where, in every winter, numerous settlements are threatened by massive glacial avalanches. On 07 Apr 12 one of the largest tragedies took place at Gyari camp in Shyok basin when a gigantic ice plus rock avalanche buried a military base and engulfed 139 persons at the spot.

RAMMS model is used for assessment of glacial hazard in the current study of simulation and modeling of potential hazardous glacial avalanches. The Remote Sensing techniques were used in the study for determination of glacial hazard coupled with ArcGIS software for geospatial analysis in the Shyok basin. The RAMMS model was firstly calibrated over the extents of Gyari glacier avalanche actual happening while it was utilized thereafter for the other potentially hazardous glaciers within the whole of the study area.

### Gyari glacial avalanche

**Figure 3:**
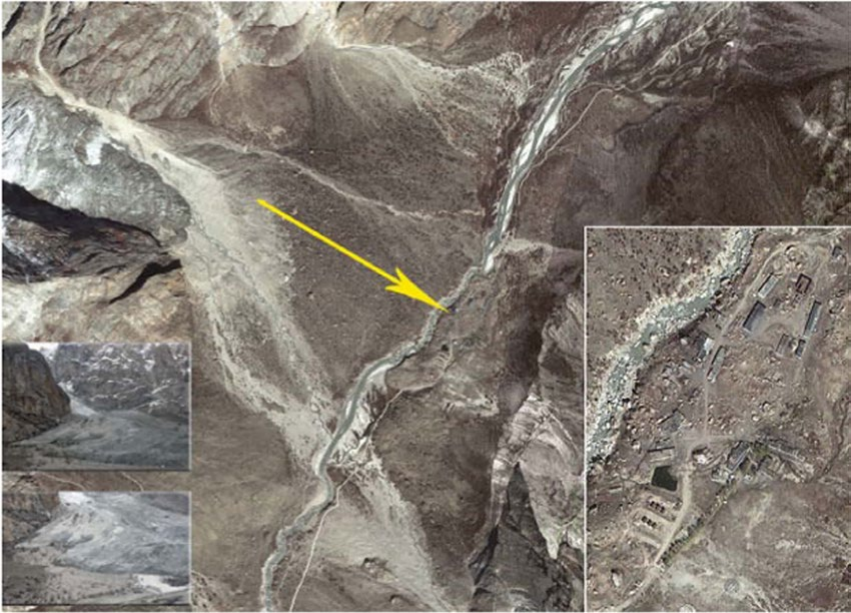
Pre disaster Gyari camp site vis-à-vis glacier. Image captured from Google Earth.

Keeping in view the investigation of the analysis performed through satellite imagery, geo-morphological and climatological conditions, it was determined that the Gyari glacier ice plus rock avalanche generation was because of the host of factors (Fig. 3).

#### Satellite imagery analysis

The pre-disaster satellite imagery of Geo Eye revealed that the melted water getting into the crevasses of Gyari glacier had generated sinkholes in the part of the glacier. So in fact because of the meteorological conditions, the part of glacier detached from the main body actually triggered the avalanche. The temporal analysis of the satellite imagery revealed that where the stress started to build up, the crevasses were clearly identified which ultimately caused detachment of glacier. Whereas satellite image of pre-disaster revealed that the max stress was built up causing max crevasses at the spot clearly shown in Fig. 3. In the year 2012, the crevasses were actually filled with loose snow which ultimately melted into water, thereby percolating into the base of the glacier and acted as a sliding lubricant.

#### Geo morphological analysis

The Geo Morphological analysis of the area resulted that the Gyari glacier didn’t have uniform topography. From 6000 m height of Gyari glacier, a downward slope of more than 45° was determined. In middle, a flat region at the center of the glacier was found. Coming from the top of the glacier, in the center region, all the mass got accumulated. Near the glacier terminus again the steepness of slope more than 35° was found. The part of the glacier detached from the lower steep slope portion of the glacier is as shown in Fig. 3.

#### Climatological analysis

Climatic variables like precipitation and temperature of past years were analyzed for any anomaly. The temperature of the Shyok basin ranges from −14.94 °C to −34.40 °C. At an elevation of 3000 to 4500 m within the basin the precipitation level ranged from 1606.04 to 1975.89 mm and at an elevation of more than 4500 m, recorded precipitation was 2482.06 mm. Gyari glacier elevation varied from 4000 to 5000 m, meaning that the max accumulation occurred at the glacier within this range of elevation. As of the year 2012, the massive accumulation of snow accounted for an unstable mass.

If climatological and geomorphological variables were recorded in time and preventive measures were taken in time, coupled with remote sensing techniques the Gyari glacier avalanche event could have been prevented.

### Calibration and validation of RAMMS model

The RAMMS model calibration was done using the Gyari glacial avalanche debris area extent using the post-disaster satellite imagery and above ground elevation of the avalanche debris. Actually no data was available in terms of on-ground identification of release area due to post-disaster heavy snowfall.

**Figure 4:**
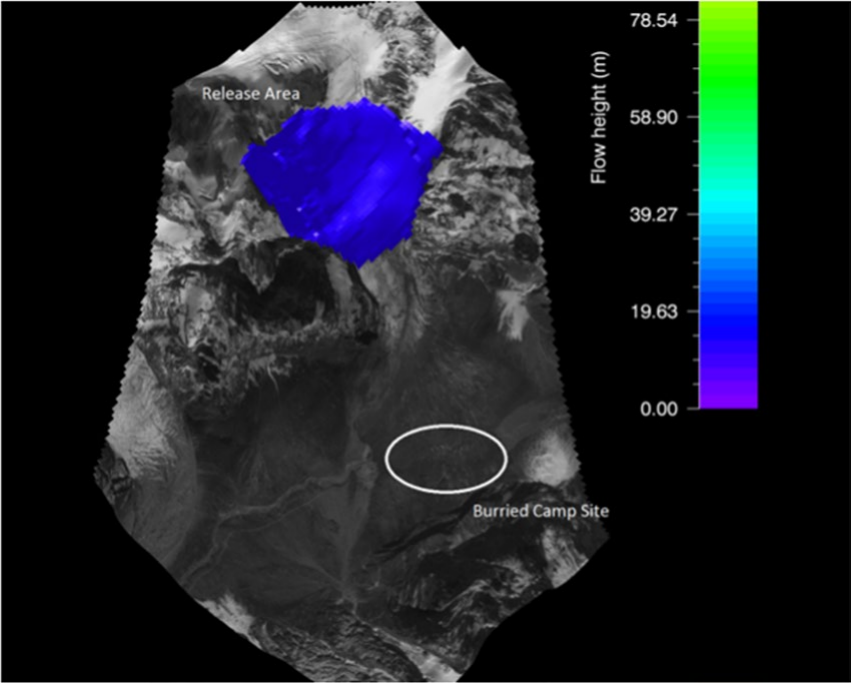
Release area of Gyari glacier avalanche (Post-disaster) vis-à-vis buried camp site. Image captured from Google Earth utilized in RAMMS model version 1.7.0 (http://ramms.slf.ch).

The RAMMS input model parameters like release area and its height were put in and simulated many times to actualize the height and debris 2D extent (Fig. 4). The RAMMS was very precisely calibrated through extensive iterative process of input parameters. The RAMMS simulated debris outline of Gyari glacier avalanche represented the actual on ground debris extent.

The RAMMS simulated 45 m as the debris height while on-site assessment it was 40 m. This difference in prediction was acceptable as of 5 m. The max modeled height for glacier avalanche No. 506 using VS model Vallee de la Sionne was determined as 41 m^15^.

### RAMMS simulations of gyari glacier avalanche

A lot of work is done to emerge a graphical user interface (GUI) for RAMMS model which made it quite easy to use. A graphical view of mathematical calculations is essential to assess the goodness of simulated outputs. One can quickly assess the modeled avalanche run-out distances, topographical features, flow velocities, entrainment depths, and flow heights.

The RAMMS model creates an output data log file which was certainly loaded with GUI afterward the accomplishment of mathematical calculation.

#### RAMMS flow height of gyari glacier avalanche

**Figure 5:**
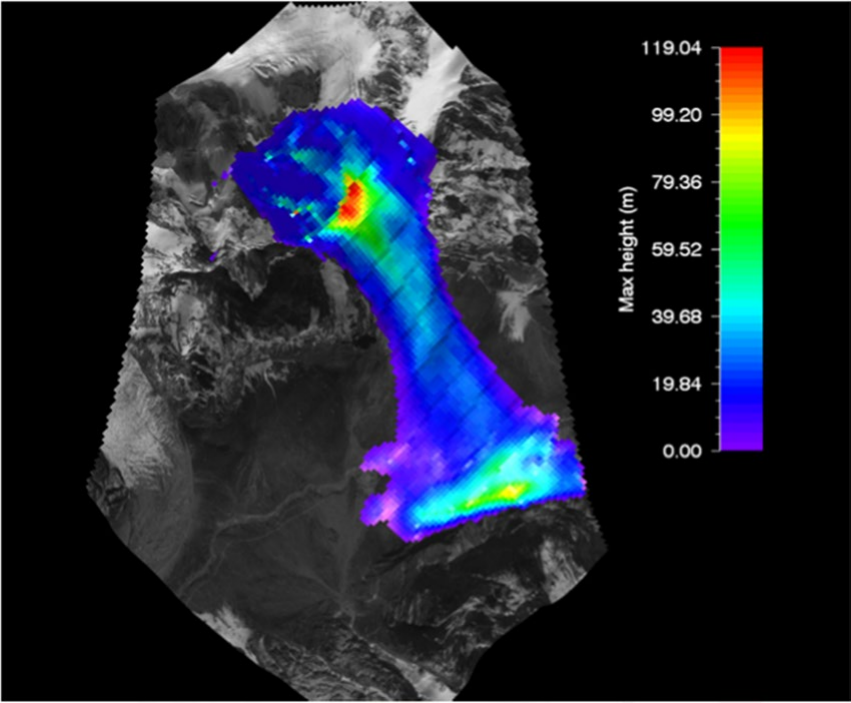
Max height of Gyari glacier avalanche. Image captured from Google Earth utilized in RAMMS model version 1.7.0 (http://ramms.slf.ch).

The output of modeled glacier avalanche flow height for Gyari glacier avalanche was ranged from 10 m (min) to 40 m (max) which was superimposed on the topography i.e. post-disaster satellite image as shown in Fig. 5.

The similar flow height of other glacier avalanches was also achieved by other researchers like Bartelt and McArdell in 2009. The topography can be portrayed on flat 2D or completely in 3D. Both dimensional representations are zoomed, moved and swapped with the purpose to find viewpoint as the best. The overlaid output is then envisioned at every step for complete simulation saved through defined steps. The color profiles of modeled flow heights of glacier avalanche allow a ready reckoner type determination of flow heights being entrained in front or tail of the avalanche at gullies or run out zones track sections.

#### RAMMS flow velocity of gyari glacier avalanche

**Figure 6:**
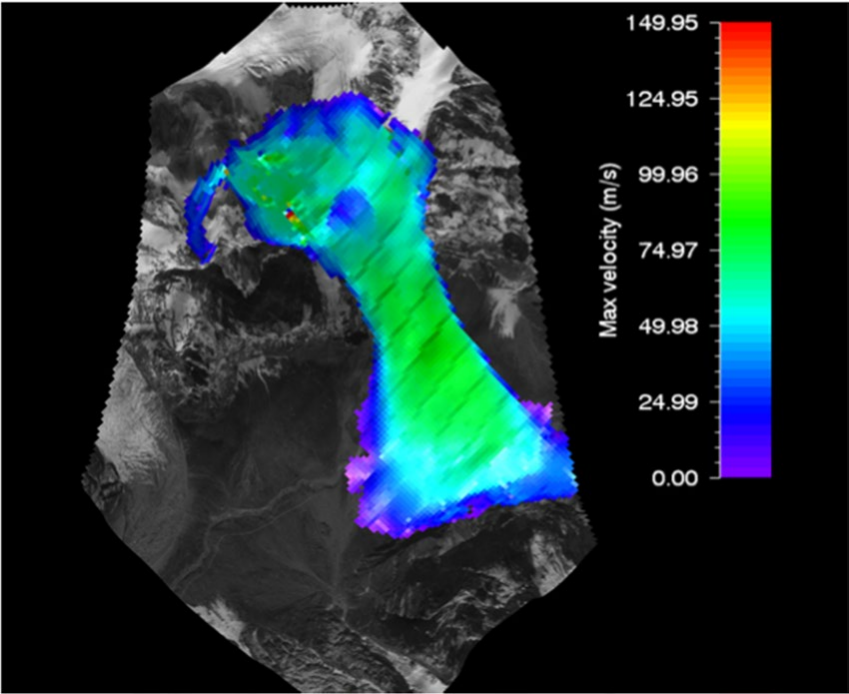
Max velocity of Gyari glacier avalanche. Image captured from Google Earth utilized in RAMMS model version 1.7.0 (http://ramms.slf.ch).

The max flow velocity of 51.8 m/s was achieved by Toinihc glacier avalanche in 2003 which was the max velocity achieved by a glacier avalanche. In 2004 the Dabanamas glacier avalanche achieved a flow velocity of 38.2 m/s. The max flow velocity for Gyari glacier avalanche molded by RAMMS was 90 m/s, a bit higher as compared to other flow velocities; this could be due to the difference in the slope and mass of the detached glacier slab. The slope and mass of the detached glaciers affect the flow velocity; higher the slope, higher would be the flow velocity and more mass of detached glacier would generate more flow velocity. The avalanche flow velocity simulated by RAMMS model for Gyari glacier avalanche was superimposed on the topography i.e. post-disaster satellite image as shown in Fig. 6.

Similarly, the topography can be portrayed on flat 2D or 3D. The color profiles of modeled flow velocity of glacier avalanche allow a ready reckoner type determination of flow velocity being entrained in front or tail of the avalanche at gullies or run out zones track sections.

#### RAMMS flow momentum of gyari glacier avalanche

**Figure 7:**
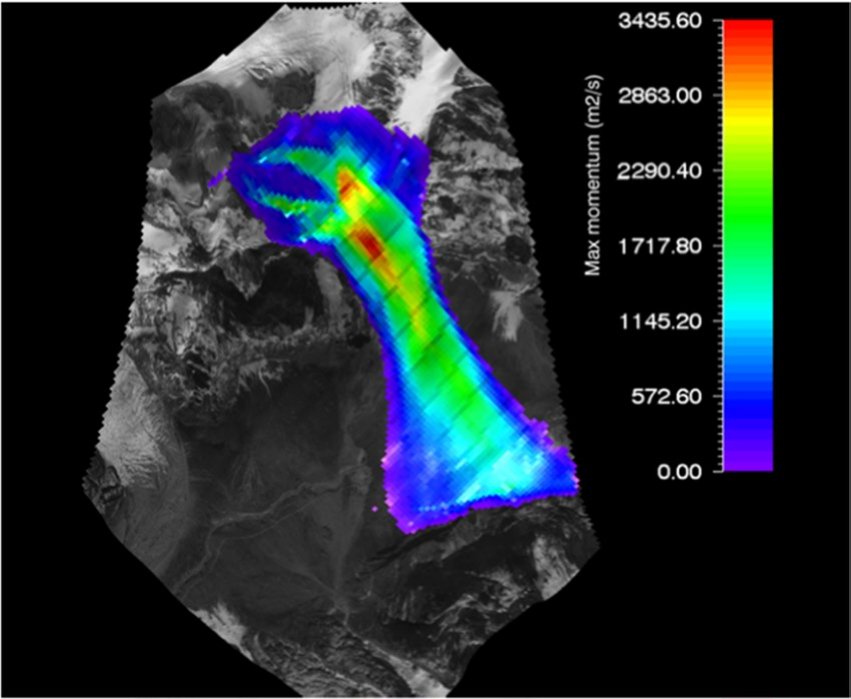
Max momentum of Gyari glacier avalanche. Image captured from Google Earth utilized in RAMMS model version 1.7.0 (http://ramms.slf.ch).

RAMMS model avalanche flow momentum for Gyari glacier avalanche was ranged from a value of 500 *m*^2^*/s* to 3500 *m*^2^*/s*. Later the momentum output was superimposed on the topography i.e. post-disaster satellite image as shown in Fig. 7.

Similarly, the topography is portrayed on flat 2D or completely in 3D. The color profiles of modeled flow momentum of glacier avalanche allow a ready reckoner type determination of flow momentum being entrained in front or tail of the avalanche at gullies or run out zones track sections.

#### RAMMS flow pressure of gyari glacier avalanche

**Figure 8:**
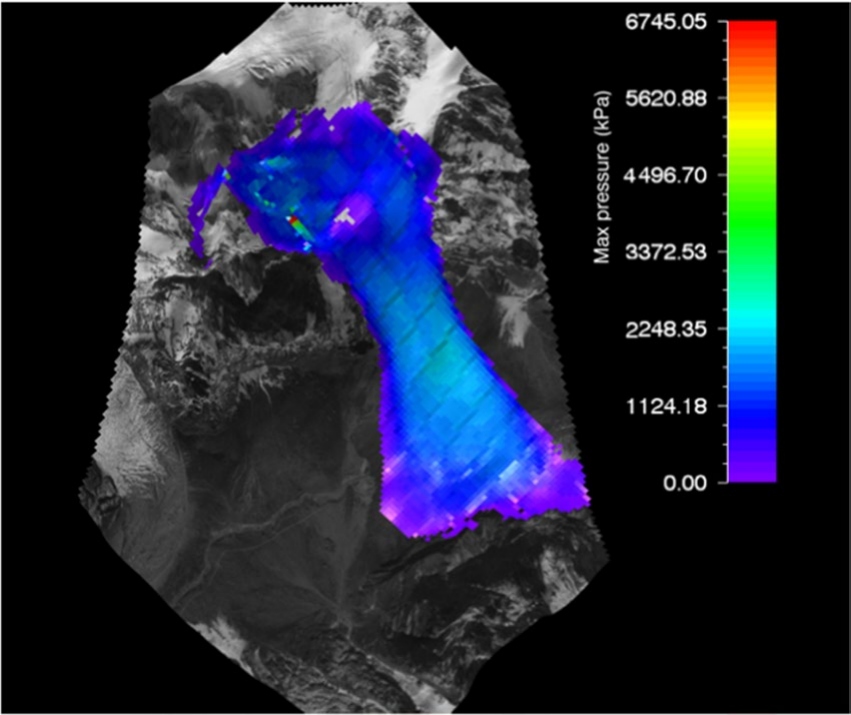
Max pressure of Gyari glacier avalanche. Image captured from Google Earth utilized in RAMMS model version 1.7.0 (http://ramms.slf.ch).

The max flow pressure output resulted by RAMMS for Gyari glacier avalanche was 2500 KPa which was superimposed on the topography i.e. post-disaster satellite image as shown in Fig. 8.

Similarly, the topography can be portrayed in flat 2D or completely in 3D. The pressure determined for avalanche No. 506 glacier by VS model for Vallee de la Sionne was 804 kPa^16^. This difference in the pressure could be attributed to the mass and slope of the slab avalanche.

The color profiles of modeled flow pressure of glacier avalanche allow a ready reckoner type determination of flow pressure being entrained in front or tail of the avalanche at gullies or run out zones track sections.

### RAMMS output parameters of gyari glacier avalanche

RAMMS model outputs coupled with the ArcGIS environment revealed better interpretation and improved spatial analysis of the modeled results of the simulation. The output parameters of flow velocity, flow height, flow pressure and flow momentum of Gyari glacier avalanche are shown in Table 3.

**Table 3:** RAMMS output parameters of Gyari glacier avalanche.

Output Parameter	Value Obtained	Description
Flow Height	10–40 m	Flow height obtained during the course of avalanche
Velocity Generated	30–90 m/s	Velocity of moving mass of avalanche
Pressure Exerted	1000–2500 KPa	Thrust Pressure of avalanche
Momentum Gained	500–3000 m^2^/s	Volumetric momentum of glacial mass
Damage Extent	600 m	Extent of damage by glacial mass of avalanche

### Potentially dangerous glaciers in shyok basin

The RAMMS model was executed for the potentially dangerous glaciers found in Shyok basin. Avalanches were simulated for all the potentially dangerous glaciers present in the Shyok basin and the log file of each glacier has shown the results of the simulations for avalanches flow velocity, flow heights, flow pressure and flow momentum.

### RAMMS simulations of shyok glacier avalanche

**Figure 9:**
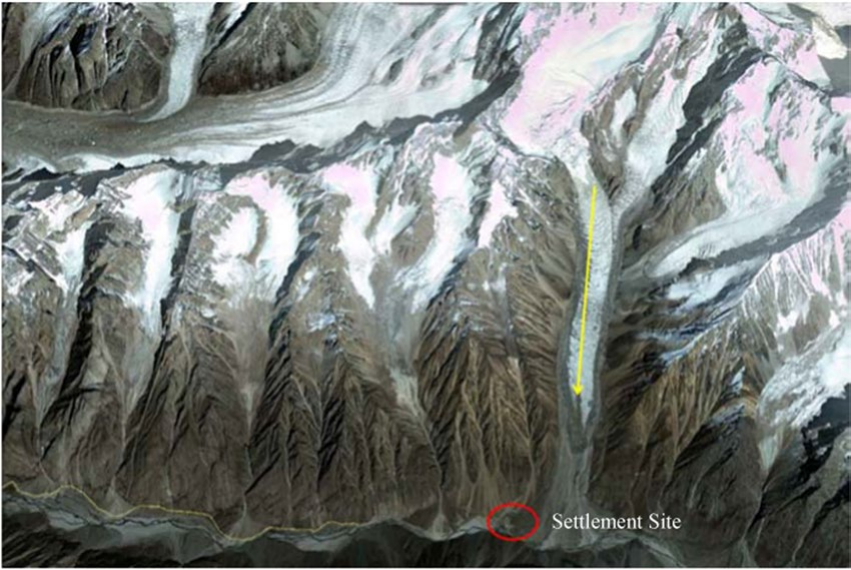
Settlement site vis-à-vis Shyok glacier. Image captured from Google Earth.

As of investigation originated from the interpretation of satellite imagery, geomorphological, climatological and the proximity analysis of the potentially dangerous glaciers, it was revealed that Shyok glacier is prone to generation of an avalanche (Fig. 9).

A lot of work is done to emerge a graphical user interface (GUI) for RAMMS model which made it quite easy to use. A graphical view of mathematical calculations is essential to assess the goodness of simulated outputs. One can quickly assess the modeled avalanche run-out distances, topographical features, flow velocities, entrainment depths, and flow heights.

**Figure 10:**
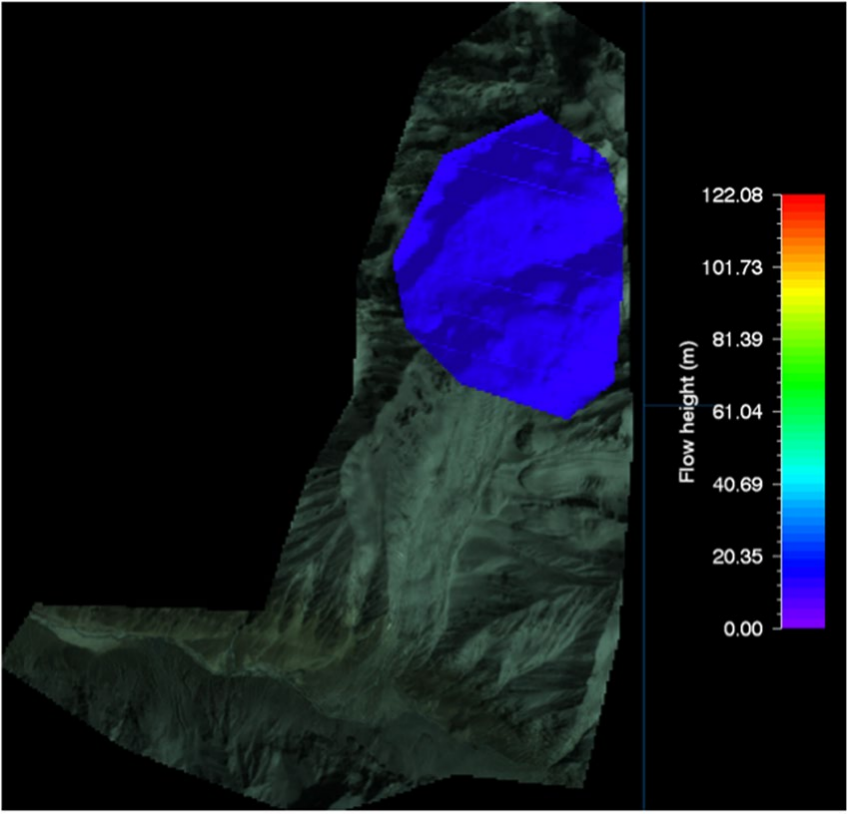
Release area of Shyok glacier avalanche (Post-disaster). Image captured from Google Earth utilized in RAMMS model version 1.7.0 (http://ramms.slf.ch).

The RAMMS input model parameters like release area and its height were put in and simulated many times to actualize the height and debris 2D extent (Fig. 10).

The RAMMS model creates an output data log file which was certainly loaded by the GUI afterward the accomplishment of the mathematical calculation.

#### RAMMS flow height of shyok glacier avalanche

**Figure 11:**
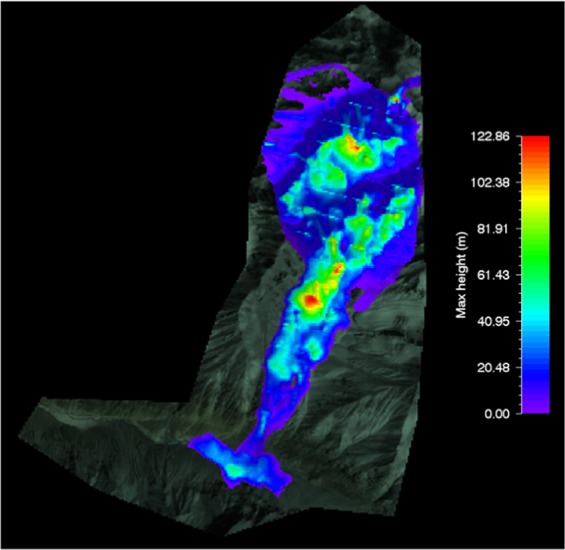
Max height of Shyok glacier avalanche. Image captured from Google Earth utilized in RAMMS model version 1.7.0 (http://ramms.slf.ch).

The output of modeled glacier avalanche flow height for Shyok glacier avalanche was ranged from 20 m (min) to 60 m (max) which was superimposed on the topography i.e. post-disaster satellite image as shown in Fig. 11.

The topography can be portrayed in flat 2D or completely in 3D. The overlaid output is envisioned at every step for complete simulation saved through defined steps. The color profiles of modeled flow heights of glacier avalanche allow a ready reckoner type determination of flow heights being entrained in front or tail of the avalanche at gullies or run out zones track sections.

#### RAMMS flow velocity of shyok glacier avalanche

**Figure 12:**
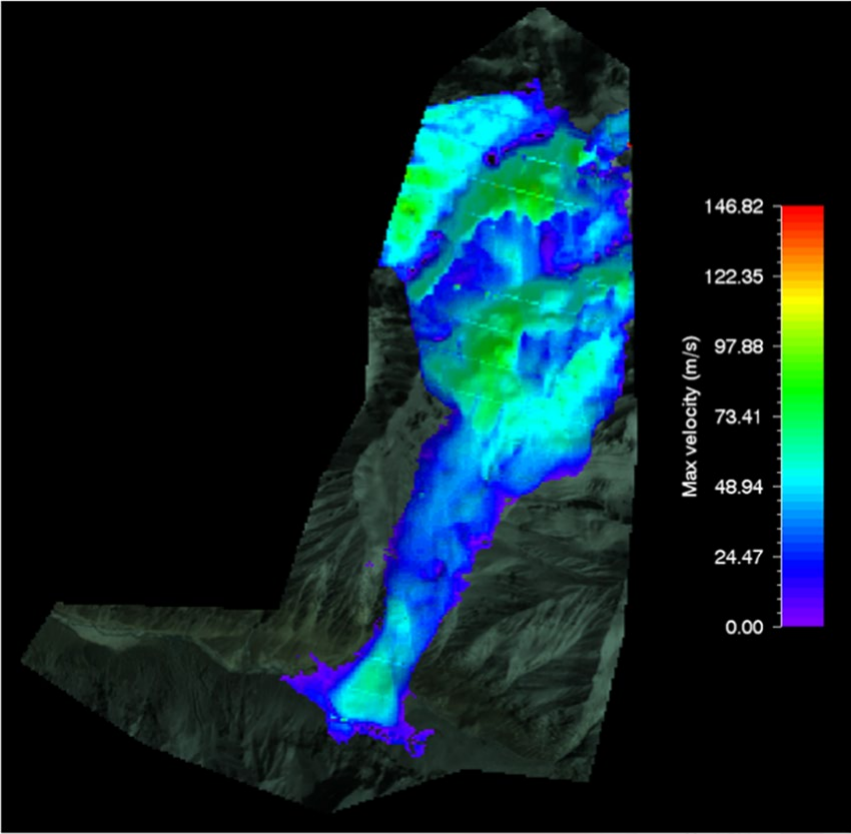
Max velocity of Shyok glacier avalanche. Image captured from Google Earth utilized in RAMMS model version 1.7.0 (http://ramms.slf.ch).

The RAMMS model simulated the velocity of 75 m/s for Shyok glacier avalanche. The max flow velocity for Shyok glacier avalanche molded by RAMMS model was a little bit high as compare to other flow velocities; this could be due to the difference in the slope and mass of the detached glacier slab. The avalanche flow velocity simulated by RAMMS model for Shyok glacier avalanche was superimposed on the post-disaster satellite image as shown in Fig. 12.

Similarly, the topography can be portrayed on flat 2D or 3D. The color profiles of modeled flow velocity of glacier avalanche allow a ready reckoner type determination of flow velocity being entrained in front or tail of the avalanche at gullies or run out zones track sections.

#### RAMMS flow momentum of shyok glacier avalanche

**Figure 13:**
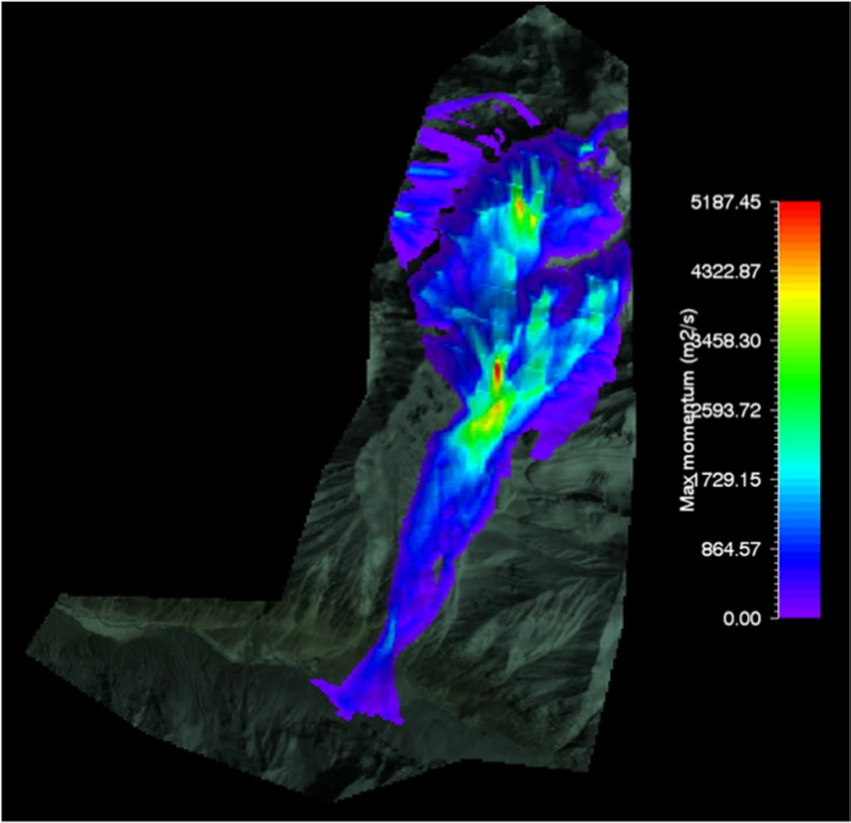
Max momentum of Shyok glacier avalanche. Image captured from Google Earth utilized in RAMMS model version 1.7.0 (http://ramms.slf.ch).

Avalanche flow momentum modeled by RAMMS for Shyok glacier avalanche was ranged from a value of 800 *m*^2^*/s* to 2800 *m*^2^*/s*. Later the momentum output was superimposed on the topography i.e. post-disaster satellite image as shown in Fig. 13.

Similarly, the topography can be portrayed in flat 2D or completely in 3D. The color profiles of modeled flow momentum of glacier avalanche allow a ready reckoner type determination of flow momentum being entrained in front or tail of the avalanche at gullies or run out zones track sections.

#### RAMMS flow pressure of shyok glacier avalanche

**Figure 14:**
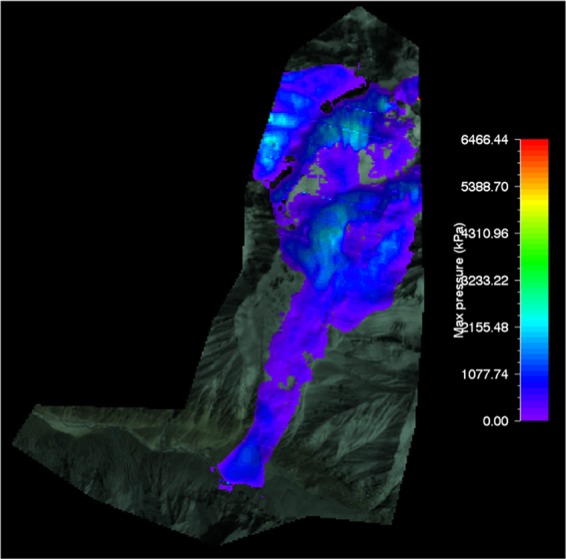
Max pressure of Shyok glacier avalanche. Image captured from Google Earth utilized in RAMMS model version 1.7.0 (http://ramms.slf.ch).

RAMMS model avalanche flow pressure simulation for Shyok glacier avalanche was 2500 KPa which was superimposed on the topography i.e. post-disaster satellite image as shown in Fig. 14.

Similarly, the topography is portrayed on flat 2D or completely in 3D. The color profiles of modeled flow pressure of glacier avalanche allow a ready reckoner type determination of flow pressure being entrained in front or tail of the avalanche at gullies or run out zones track sections.

### RAMMS output parameters of shyok glacier avalanche

RAMMS model outputs coupled with the ArcGIS environment revealed better interpretation and improved spatial analysis of the output results of the simulation. Model output parameters of flow velocity, height, pressure, and momentum of Shyok glacier avalanche are shown in Table 4.

**Table 4:** RAMMS output parameters of Shyok glacier avalanche.

Output Parameter	Value Obtained	Description
Flow Height	20–60 m	Flow height obtained during the course of avalanche
Velocity Generated	25–75 m/s	Velocity of moving mass of avalanche
Pressure Exerted	1000–2500 KPa	Thrust Pressure of avalanche
Momentum Gained	800–2800 m^2^/s	Volumetric momentum of glacial mass
Damage Extent	700 m	Extent of damage by glacial mass of avalanche

## Conclusions

The RAMMS model is an effective tool for simulation and modeling of glacier avalanche hazard. The primary results of the application of RAMMS model for simulation of Shyok basin glacial avalanches indicate that RAMMS closely reproduces max flow velocities, heights, pressures, momentums and run out distances in 2D and 3D terrain precisely. Flow widths were well modeled for the starting and transit zones by using statistical parameters utilized by many experts in glacier avalanche assessment. Input parameters values are established through a calibration process of debris extent matching of modeled output and actual debris extent of Gyari avalanche. The analysis is based on DEM in order to identify in between the channel, gully regions and open slopes. The simulation results of the RAMMS model were promising. RAMMS model is effectively used for hazard assessment studies as the results are quite close to reality, especially because it contains both RKE model and VS model features preserving many of the features of these models and can precisely predict the spatial distribution of the avalanche debris deposits. The RAMMS simulation analysis revealed that Gyari base camp was situated in the course of glacier avalanche and was an avalanche hazard prone area. Moreover, by incorporating the RAMMS simulation results, the settlements near the other identified glacier avalanche hazard-prone areas can be safeguarded to avoid the destructive incident like Gyari in future.
